# Comparative Analysis of Integrated Filtering Methods Using UWB Localization in Indoor Environment

**DOI:** 10.3390/s24041052

**Published:** 2024-02-06

**Authors:** Rahul Ranjan, Donggyu Shin, Yoonsik Jung, Sanghyun Kim, Jong-Hwan Yun, Chang-Hyun Kim, Seungjae Lee, Joongeup Kye

**Affiliations:** 1 Department of Computer and Electronic Convergence, Intelligent Robot Research Institute, Sun Moon University, Asan 31460, Republic of Korea; rahulroboticskr@gmail.com; 2 Department of Computer Engineering, Intelligent Robot Research Institute, Sun Moon University, Asan 31460, Republic of Korea; rlsl0422@sunmoon.ac.kr (D.S.); dbstlr0225@sunmoon.ac.kr (Y.J.); 3 Department of Mechanical Engineering, Kyung Hee University, Suwon 17104, Republic of Korea; kim87@khu.ac.kr; 4 Mobility Materials-Parts-Equipment Centre, Kongju National University, Kongju 32588, Republic of Korea; yunjh0915@kongju.ac.kr; 5 Department of AI Machinery, Korea Institute of Machinery and Materials, Daejeon 34103, Republic of Korea; chkim78@kimm.re.kr; 6 Department of Mechanical Engineering, Sun Moon University, Asan 31460, Republic of Korea; jekye@sunmoon.ac.kr

**Keywords:** ultra-wideband (UWB), moving average filter (MVG), Kalman filter (KF), extended Kalman filter (EKF), robot operating system (ROS), LiDAR, robot navigation

## Abstract

This research delves into advancing an ultra-wideband (UWB) localization system through the integration of filtering technologies (moving average (MVG), Kalman filter (KF), extended Kalman filter (EKF)) with a low-pass filter (LPF). We investigated new approaches to enhance the precision and reduce noise of the current filtering methods—MVG, KF, and EKF. Using a TurtleBot robotic platform with a camera, our research thoroughly examines the UWB system in various trajectory situations (square, circular, and free paths with 2 m, 2.2 m, and 5 m distances). Particularly in the square path trajectory with the lowest root mean square error (RMSE) values (40.22 mm on the *X* axis, and 78.71 mm on the *Y* axis), the extended Kalman filter with low-pass filter (EKF + LPF) shows notable accuracy. This filter stands out among the others. Furthermore, we find that integrated method using LPF outperforms MVG, KF, and EKF consistently, reducing the mean absolute error (MAE) to 3.39% for square paths, 4.21% for circular paths, and 6.16% for free paths. This study highlights the effectiveness of EKF + LPF for accurate indoor localization for UWB systems.

## 1. Introduction

Wireless sensor network (WSN) localization and positioning systems have become more and more popular in the past few years. They have been effectively applied in a number of different applications, including hospital equipment localization and warehouse product tracking [1]. The particularities of indoor channels necessitate precise and economical estimation schemes for indoor positioning systems. There is not a universal indoor positioning system, in contrast to outdoor positioning systems like the Global Positioning System (GPS). As a result, a well-designed solution that takes into account the energy limitations, low infrastructure cost, and limited capacity of a wireless sensor network (WSN) is needed [2]. Each year, the tragedy of indoor emergency incidents causes great shocks to society and leaves families with unimaginable losses. This is primarily because emergency situations, such as fires, present a hazardous and smoke-filled environment. Localization is one of the most directional problems, since it is hard to locate the person who is trapped in the building without knowing where they are. Not only is localization crucial for evacuees looking for outside assistance, it is also crucial for self-evacuation. Unfortunately, due to issues with accuracy, cost, and efficiency, a practical and trustworthy indoor localization system is not currently available [3].

For indoor positioning systems, various techniques have been used, such as measuring the distance or range between the target and the anchor sensor using methods such as the angle of arrival (AOA), received signal strength (RSS), time of arrival (TOA), time difference of arrival (TDOA), and time of flight (TOF). TOF, which averages the round-trip time of packets, is a promising low-cost solution for real-time applications. Trilateration, location fingerprinting, and proximity algorithms are all designed to calculate position based on distance measurements. Trilateration is preferred due to its ease of use and high processing speed [4].

Ultra-wideband (UWB) technology is a wireless communication method characterised by high data rates, low power consumption, and precise ranging. When compared to traditional technologies like Wi-Fi, UWB stands out for its ability to provide fine spatial resolution, resistance to multipath interference, and high accuracy in indoor environments [5]. While Wi-Fi and Bluetooth Low Energy are popular indoor positioning technologies, UWB has distinct advantages in terms of accuracy and interference resistance. RFID and ultrasonic technologies, while useful in some applications, frequently fall short of UWB’s precision and reliability.

However, measurement noise reduces the accuracy of position estimation. Modelling radio propagation and time delay for WSNs in indoor environments presents challenges that lead to measurement errors and data loss due to factors such as low signal-to-noise ratio (SNR), severe multipath effects, reflections, and link failures. To address these issues, the KF has been implemented in WSN systems. KF, a recurve linear filtering model, is commonly used to estimate tracks from noisy measurements. It is effective at smoothing random deviations from the true target path. Despite its advantages, KF is still susceptible to errors when measurement noise is high. To reduce noise, the distributed Kalman filter has been proposed, but it requires global information, which is not feasible in real-world positioning systems where sensors can only provide partial range values [6]. RADAR systems use KFs but do not take environmental changes into account, making them unsuitable for real-world applications [7]. Furthermore, KF assumes that noise has an additive white Gaussian distribution and is linear, which is difficult to model in indoor environments where wireless channels change frequently due to object movement and surface reflections.

Section 2 begins with a thorough review of previous research in the field of indoor localization and filtering algorithms, as well as the paper’s contribution. We examine the advantages and disadvantages of existing approaches and show how the proposed method addresses some of these issues. Section 3 delves into the methodology employed, specifically the system architecture entailing the integration of ultra-wideband (UWB) technology with a visual tracking system. The ROS ecosystem and flowchart are then explained in Section 4. Section 5 introduces the proposed method for accurate localization, which is at the heart of the research. The method includes MVG, KF, and EKF, as well as a novel integrated model with UWB sensors. The integrated model is intended to improve the accuracy and reliability of localization [8]. Section 6 presents detailed information on the experimental setup and the various components used during the evaluations to provide practical insights. Finally, Section 7 concludes the paper by discussing the obtained data results and potential future research directions. We emphasize the importance of improving robotic localization accuracy and loss using UWB sensors, paving the way for future advancements in this field.

## 2. Related Works

Localization systems that use ultra-wideband (UWB) signals estimate the position of an object or person in an environment. UWB signals have some advantages for localization, including high accuracy, resolution, and data rate, as well as multipath resistance. However, noise, interference, non-line-of-sight (NLOS) propagation, and high time resolution are some of the challenges that UWB localization systems face [9].

To address these challenges and improve the localization accuracy and robustness of UWB systems, various algorithms have been proposed. Some common methods are based on measurements of time-of-arrival (TOA), angle-of-arrival (AOA), or phase-difference-of-arrival (PDoA). These methods are further classified as deterministic or probabilistic based on whether they estimate position using geometric or statistical models. MVG, KF, and EKF are deterministic approaches that are widely used to reduce noise and interference effects on UWB measurements. However, in NLOS scenarios where UWB signals are obstructed by obstacles and reflect from multiple paths [10,11,12], these algorithms may not perform well.

The particle filter (PF), Bayesian filter (BF), and support vector machine (SVM) are popular probabilistic approaches for dealing with the NLOS problem by incorporating prior knowledge or learning from data [13,14]. These algorithms, however, may suffer from high computational complexity or poor generalization ability. The integrated model entails applying LPF to raw UWB data before passing them on to the MVG, KF, or EKF algorithms [15]. The LPF soothes the UWB data and reduces the effects of high-frequency noise and interference. Based on the filtered UWB data, the MVG, KF, and EKF algorithms are used to further reduce noise and interference effects and estimate position [16].

Thus far, we have explored different types of indoor localization systems. While these systems have reduced localization errors and solved localization challenges, further positioning accuracy improvements are needed to improve indoor localization and accuracy. This paper seeks to address the above issues through the following contributions:

1. This research enhances the development of UWB (ultra-wideband) localization systems by building on previous studies and suggesting innovative integrated filtering techniques for indoor localization. The investigation examines the use of moving average filter (MVG), Kalman filter (KF), and extended Kalman filter (EKF) algorithms, along with a pioneering integrated filtering strategy integrating a low-pass filter (LPF) into MVG, KF, and EKF. Our work aims to enhance solutions for improving UWB localization accuracy.
2. This integration aims to enhance accuracy and minimize noise in filtering algorithms. Consequently, our approach effectively reduces high-frequency inference and noise. In contrast to existing probabilistic techniques such as particle filter, Bayesian filter, and support vector machine, which exhibit computational complexity and limited generalization ability, our proposed model is mathematical. This eliminates the need for computational complexity or training, allowing for easy generalization to various paths, including square, circular, and free paths.

This study goes further than the precise algorithms used in this paper, concentrating on how our filtering techniques combine towards the wider context of UWB localization. The proposed methodologies present a hopeful route to tackle current issues and enhance the precision and dependability of indoor tracking and positioning technologies.

## 3. Working Methodology

This section describes the architecture of the ultra-wideband (UWB) system, the vision tracking system, and the ROS ecosystem. The architecture of the UWB system is intended to enable robot positioning and localization. To determine the ground truth, the vision tracking system employs camera vision techniques to track and monitor the movement of the robot [17,18]. ROS 1.15.14 Noetic is a software framework that allows software and hardware to communicate, thereby providing tools for building, testing, and deploying in localization.

### 3.1. System Architecture

**Figure 1:**
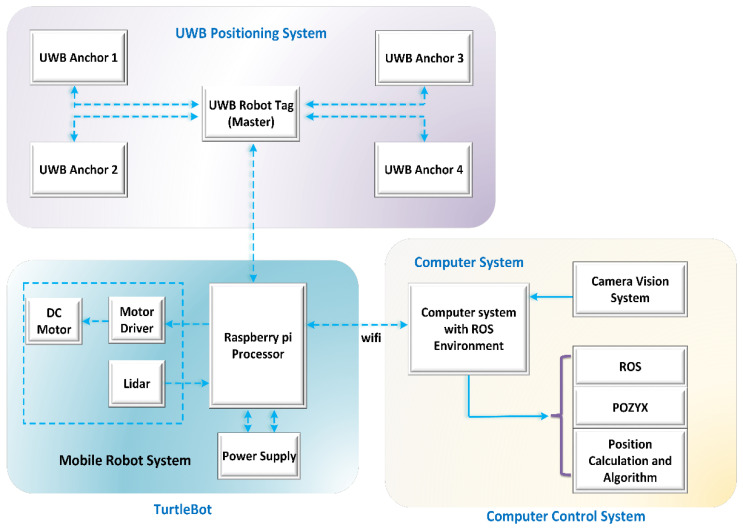
Working architecture.

The robot positioning system, as shown in Figure 1, is made up of a UWB positioning subsystem, a remote computer, and a mobile robot. The UWB positioning subsystem is made up of UWB anchors that are permanently attached to the environment and a UWB robot tag [19]. The computer executes positioning algorithms to determine the robot’s position and coordinates by measuring the distance between the UWB tag and the anchors. The remote computer communicates with the robot wirelessly, processes positioning data, computes the robot’s coordinates, and controls its movement. The system is capable of a variety of tasks, including interactive communication, robot control, position estimation, and robot position display. Due to errors or instability, a single sensor cannot achieve high accuracy. The use of UWB tags in conjunction with anchor sensors can improve positioning accuracy and stability. A position estimation and error correction method based on the EKF algorithm was proposed in this study. As shown in Figure 1, the robot positioning system can acquire and integrate data from UWB tags and anchors at the same time.

The proposed system architecture is composed of three major blocks: UWB positioning, mobile robot, and computer control systems. The first block is made up of several UWB tags and anchors. The robot development system, which is a mobile robot system, is the next block. The mobile robot for the first position experiment was TurtleBot 3. A motor for locomotion, a driver unit for controlling the motor, and a LiDAR sensor for scanning and detecting obstacles make up the system. All of these systems, which are powered by a Raspberry Pi, are controlled by a Raspberry Pi processor. Finally, a computer control system with a ROS environment and POZYX library was used, which communicated via Raspberry Pi as a read/write device [20]. To improve localization and positioning, various algorithms can be tested and simulated. TurtleBot3 is outfitted with UWB tag nodes that are compatible with the ROS ecosystem. The connection between the UWB robot tag and the Raspberry Pi is made via a USB interface, as shown by the dashed line. These sensor measurements, along with wheel odometry and LPF algorithm output, are used in the EKF for localization, and the LiDAR-based navigation stack is started with UWB ranging and LiDAR scanning. Furthermore, the TurtleBot3 ROS library makes it easier to implement a simulation environment.

### 3.2. UWB System

Traditionally used for wireless communications, UWB technology has recently gained popularity in positioning applications. Because of its wide bandwidth, it is resistant to interference from other radio frequency signals and can penetrate obstacles and walls, making it a reliable positioning technology in non-line-of-sight and multipath environments [21]. Furthermore, the unique identification of tags in a UWB system solves data association problems automatically. The technology sends radio signals from a mobile transceiver (tag) to a group of known anchors (A1–A3), measures the time of flight (TOF), and calculates distances. The POZYX system, a UWB-based hardware solution for precise position and motion sensing, was used in this study. The UWB configuration settings can be customized based on four different parameters that affect system performance. However, the presence of noise and uncertainty in the measurement data collected by UWB sensors necessitates the use of advanced filtering techniques for accurate position estimation, such as KF and EKF, which can improve the accuracy of UWB-based indoor localization systems.

### 3.3. Visual Tracking System

**Figure 2:**
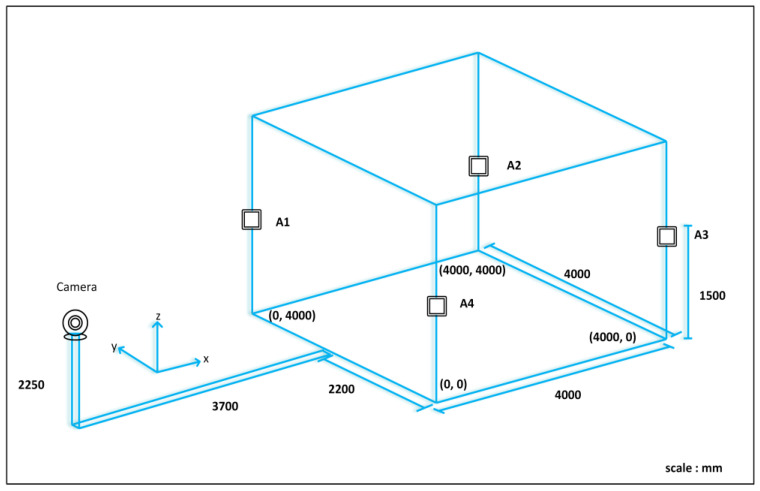
Testing environment structure.

Visual tracking is a computer vision technique that involves tracking and estimating the motion of objects in a sequence of images or video frames captured by a webcam. It is a fundamental task in many applications, including surveillance, robotics, augmented reality, and human–computer interaction [22,23]. As illustrated in Figure 2, visual tracking methods aim to accurately and robustly locate and track objects of interest despite changes in appearance, scale, orientation, and occlusion.

Figure 2 depicts a basic process for visual tracking using a webcam installed and tested at the IRRI laboratory of Sun Moon University in South Korea. We placed a webcam in an appropriate location, making sure that all corners of the tracking area on the webcam were visible. Based on the webcam capture, we captured and saved four positions in a 2D projected coordinate system. We created a perspective transformation matrix using the saved positions that maps the webcam’s view to the desired tracking area. The image matrix and perspective transformation matrix were then computed using a 512 × 512 resolution for optimal tracking performance.

The visual tracking process began after applying the perspective transformation. We selected a high-contrast area of the robot for tracking. An appropriate tracking algorithm, such as the discriminative correlation filter with a channel and spatial reliability (CSRT) algorithm, was implemented to track the robot within the 512 × 512 images [24]. The tracking data provide the position within the 512 × 512 image and are converted to the corresponding position in the real environment, which may have different dimensions. By scaling the tracked position using Equation (1)**,** the tracked position is mapped to the 4000 mm×4000 mm environment in *x* and *y*, where$\;\bar{x}\;$and $\bar{y}$ represent the positions within the 512×512 image.

(1) $$x=\frac{\bar{x}}{512}\times 4000,\;y=\frac{\bar{y}}{512}\times 4000$$

**Figure 3:**
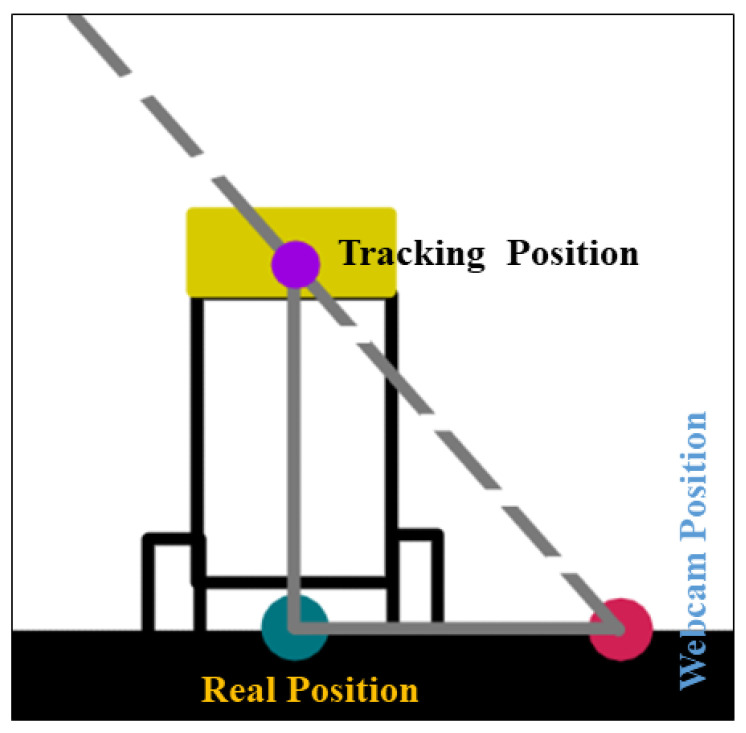
Webcam line of sight.

To ensure accuracy, it is important to eliminate any bias in the tracking system. The robot was moved to known positions, such as [1000, 2000], [2000, 2000], [3000, 2000], [2000, 1000], and [2000, 3000], and the tracking data were observed. Figure 3 shows the line of sight of the webcam, which is represented by a dotted line, and the real position, webcam position, and tracking point are represented by green, pink, and purple dots, respectively. The bias length is the distance between the green and pink dots.

If the bias is consistent along the axis, the bias is corrected using an average. If the bias has a linear pattern, a one-dimensional polynomial fit is used to estimate the bias and adjust the tracked positions. In this study, the visual tracking method was used with a webcam to provide accurate and reliable tracking information over the following steps.

The creation of the perspective transformation matrix in the process of visual tracking using a webcam involves capturing and saving four distinct positions in a 2D projected coordinate system based on the webcam capture. These positions are chosen in such a way that they cover the entire tracking area visible to the webcam. Following that, the perspective transformation matrix is generated using the saved positions. The matrix effectively maps the webcam’s view to the desired tracking area, providing a transformation that accounts for appearance, scale, and orientation variations. A combination of geometric and algebraic methods is used to calculate the elements of the transformation matrix, ensuring an accurate representation of the spatial relationship between the webcam’s view and the real-world tracking area. For optimal tracking performance, a resolution of 512 × 512 is used [25]. This meticulous process ensures that the visual tracking system can estimate the motion of objects within the designated tracking area robustly and accurately, which is a critical component of our experimental setup.

### 3.4. ROS Ecosystem

In this section, we will look at the ROS ecosystem and its components, such as nodes, topics, and messages, as well as how UWB sensors can be integrated with it. We also go over how UWB sensors are used to measure the distance between a robot and its surroundings, and how these data can be used to improve the robot’s localization and mapping capabilities.

For navigation systems, the most important information is position. Because they provide low-noise-range information that is resistant to multipath interference, UWB sensors are an excellent choice for robot localization [26]. The combination of their information and odometry data provides a robust solution for difficult environmental conditions. The POZYX system uses UWB technology to achieve centimeter-level accuracy, which is far superior to traditional Wi-Fi and Bluetooth positioning systems. The algorithm computes the robot’s position; applies the KF, which sets the motion model using odometry data; and updates the pose using UWB range measurement pose information. The map and current robot position are displayed on the GUI, and the navigation stack can be fed with KF-based pose information on demand. There are several ROS packages available for collecting and processing IMU sensor data in mobile robots, such as the ROS IMU package, which provides an implementation of an IMU sensor driver as well as a filter for estimating the robot’s orientation using sensor data [27]. Robot localization, for example, provides an implementation of EKF for fusing data from multiple sensors, including IMU data, to estimate the robot’s position and orientation [28].

In addition, we conducted an experiment using the robot operating system (ROS) and used a flexible framework for writing robot software, offering a collection of tools, libraries, and conventions that aim to simplify the task of creating complex and robust robot behavior across a wide variety of robotic platforms.

**Figure 4:**
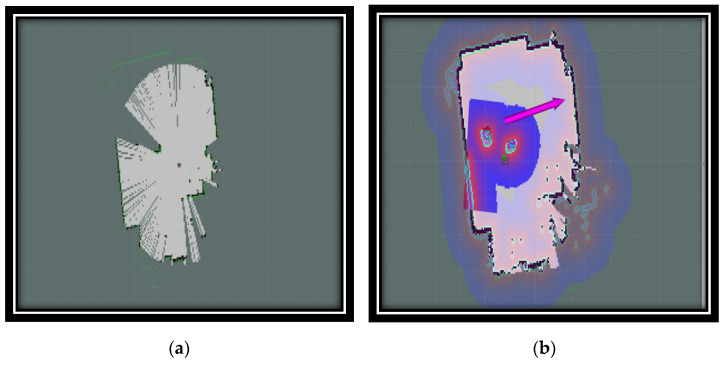
(**a**) Robot poses and LiDAR scans before the automatic initialization; (**b**) robot poses and LiDAR scans after the automatic initialization.

We used a 3D visualization tool called RViz to visualize sensor data, robot models, and robot states, among other things. Figure 4a displays a map generated by sensor data from a robot. The map representation consists of white dots forming shapes that likely represent obstacles or objects detected by the robot’s sensors. There are green lines indicating certain paths or directions related to the robot’s movement or sensor orientation.

After conducting the experiment in Figure 4b, we were able to generate a colorful map with purple, white, and green areas. We were able to see two green circles with red lines (possibly indicating the orientation) that appeared to represent robots or sensors. A purple arrow extended from one of the green circles, possibly indicating a planned path or direction of movement. The background grid suggested this was some form of spatial mapping or planning tool. Using RViz, we were able to visualize the data we collected from the experiment. We were able to see the data in a way that made them easy to understand and analyze. This helped us to draw conclusions about the data and make decisions based on our findings. Figure 4 illustrates the difference in the robot’s pose before and after the initialization process. Before autonomous initialization, the robot’s pose is incorrect and the scan data do not match the real map, as shown in Figure 4a; however, after initialization, the scan data and map are aligned, as shown in Figure 4b.

Once the robot’s starting position has been determined, the robot starts moving to point A using the “Move_Base “algorithm available in the ROS navigation stack. The final position of the robot is then determined, both on the map and in the real world, after starting with a precise initialization. The accuracy of the robot’s arrival at its destination is confirmed by analyzing the LiDAR scans on the map.

### 3.5. System Flowchart

**Figure 5:**
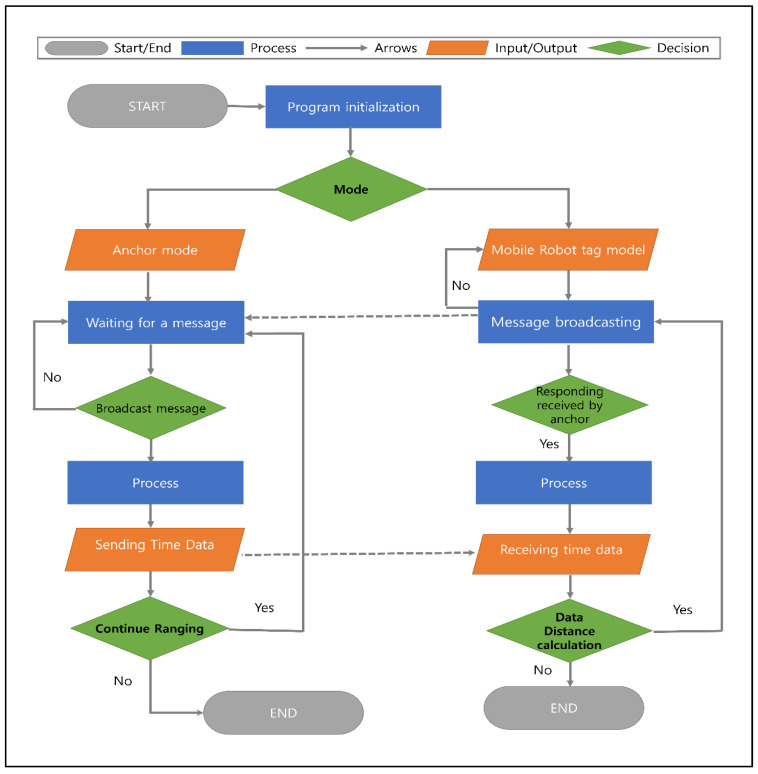
System flowchart.

An indoor UWB localization system flowchart is a visual representation of the steps and processes involved in the system’s operation. It outlines the sequence of events that occur from beginning to end, providing a comprehensive understanding of how the system operates. In this system, a flowchart depicts the entire process. For various tasks, the system provides both simulation and real-world test environments. The user has the option of running the application in a simulation or in the real world. When the application is run in simulation mode, the Gazebo and POZYX simulations are launched, and synthetic sensor and map data are obtained. Real sensor data are obtained if the application is run in the real world [29]. The initialization package then uses the UWB range and LiDAR scan data to finish the autonomous initialization process, whether the data are synthetic or real. Figure 5 depicts a system flowchart of the UWB localization system, which outlines the system’s various components and how they interact with one another.

The flowchart begins by initializing the program. The mobile tag mode or the anchor mode is selected. If the mobile tag mode is selected, the program will begin sending messages while waiting for a response from the anchor. When a response is received, the processes the data and sends time data to the tag mode as the receiving data mode. The data distance is then calculated, which involves calculating the distance between the mobile tag and the anchor.

When the anchor mode is selected, the program waits for a message from the mobile tag. Once the message is received, the program processes the data and responds to the mobile tag. The flowchart in Figure 5 shows a high-level overview of the UWB indoor localization system. It clarifies the steps involved in the operation of the system and how the different components interact to achieve the desired result.

## 4. Filtering Algorithm

In this section, we discuss the filtering algorithm for implementing the filtering process [30,31]. The filtering algorithm aims to extract relevant information from noisy or incomplete data by applying mathematical techniques.

The filtering algorithm is a crucial process in signal processing that aims to extract relevant information from noisy or incomplete data. This section discusses two types of filtering algorithms: the moving average filter and the Kalman filter. The moving average filter is a simple method used to smooth data by calculating the sampled average and eliminating noise [32]. It can be expressed as a recursive function, where the current average is calculated as a weighted sum of the previous average and the current observation.

The Kalman filter, on the other hand, is a mathematical framework that includes estimation and correction steps. It consists of a set of equations divided into two main steps: prediction (estimation equations) and correction (measurement equations). The prediction step determines the estimated value, while the correction step updates the estimated value using the latest measurement. The extended Kalman filter (EKF) is an extension of the traditional Kalman filter, which provides a recursive solution to the problem of estimating the state of a system over time. It incorporates nonlinear functions of the system state into a linear approximation, which can then be updated using a recursive Bayesian filter. The low-pass filter (LPF) is another type of filter that allows signals with low frequencies to pass through while attenuating higher frequencies. It is commonly used in signal processing to filter out high-frequency noise, as the main signal of interest often is in the lower frequency range. The LPF equation is expressed as a weighted sum of the previous estimate and the current observation, where the weighting factor determines the weight given to the previous estimate versus the current observation [33,34,35].

## 5. Proposed Algorithm

### 5.1. Low-Pass Filter and Moving Average Filter (LPF + MVG)

The “LPF + MVG” algorithm (Algorithm 1) was created especially for indoor localization applications. First, measurements are gathered from the UWB or other comparable technology, such as TOF data. MVG is used in these measurements in order to increase accuracy and decrease noise. Next, using the reference point locations and processed TOF data, trilateration is used to estimate the target’s position.
**Algorithm 1** LPF + MVG 1: 2: 3: 4:**Input: data = **${[(x}_{raw}$,$\;y_{raw)},\;(x_{truth},\;y_{truth})]$ LPF–Averaging filter (data) filtered_data = [] **For i in range (len(data));**5: Measurement (TOF ());6: Lateration ();7: LPF–Averaging filter ()8: 
**{**9: 

**if** no valid data, then10: 


return state: link failure or system failure11: 

**else**12: 13: 14: 15: 


Averaging (); ∖∖calculate the expected value of the range average_value = np.mean(data[start:end]) filtered_data = filtered_data.append(average_value) return: filtered_data16: 

**end if**17: 
**}**18: Lateration ();19: LPF–Averaging filter ();20: Print (filtered_data)21: **End for**

In order to improve the results and remove noise from the position estimates, the algorithm adds an LPF + MVG. The LPF + MVG algorithm combines these filtering methods to strike a compromise between responsiveness and noise reduction, guaranteeing precise and trustworthy position estimates even in intricate indoor settings. To smooth the trajectories, this algorithm maps the robot’s input position to the ground truth position based on the LPF + MVG from the robot’s data point.

### 5.2. Low-Pass Filter and Kalman Filter (LPF + KF)

The localization algorithm LPF + KF (Algorithm 2) makes use of indoor region data, like room size, to adjust measurements. It functions as an integrated filter and exhibits KF behavior when the data are inside the indoor region’s bounds. Here, it uses steps for prediction and correction to calculate positions. However, the system acts like a low-pass filter and depends on the predicted value from the prior state when data go outside of the bounds (or when the system comes across disturbance information). The LPF + KF system has the ability to provide precise and trustworthy indoor position estimates. When there is a lot of measurement noise, LPF + KF usually depends on its expected value. The algorithm is especially well suited for tracking motion in a small space because this kind of motion usually travels along straightforward, simple paths at low speeds. Because of this, LPF + KF works well in situations where motion is slow and does not take convoluted or complicated paths. When processing LPF + KF, the estimated state of the robot’s position is provided, and the noise effect is also calculated. The LPF + KF will then adjust the step for the subsequent state.
**Algorithm 2** LPF + KF1: 2: 3: 4:**Input: data = **${[(x}_{raw}$,$\;y_{raw}),\;(x_{truth},\;y_{truth})]$ LPF–Kalman filter (data) filtered_data = [] **For i in range (len(data));**5: Measurement (TOF());6: LPF–Kalman filter ()7: 
**{**8: 

**if** (Positions out of bound **then**9: 

       **return**
$\left[\hat{x},\;\hat{y}\right]$
10: 

**else**11: 12: 13: 14: 15: 16: 17: 18: 19: 20: 21: 


LPF–Kalman filter (); ∖∖calculate the expected value of the range State Estimate = $\left[\hat{x},\;\hat{y}\right]$ Process Noise                                                ${\hat{x}}_{k}=x_{k}+K_{k}\left(z_{k}-Hx_{k}\right)$ Correction Step ${\;z}_{k}=H\;x_{k}+r_{k}$ Predict Step                                                ${\;x}_{k+1}=Ax_{k}+q_{k}$ filtered_data return: filtered_data22: 

**end if**23: 
**}**24: LPF–Kalman filter ();25: Print (filtered_data)26: **End for**

### 5.3. Low-Pass Filter in Extended Kalman Filter (LPF + EKF)

LPF + EKF is comparable to LPF + KF, with the exception of algorithm trilateration. Instead of calculating measurement positions, this algorithm, called LPF + EKF, uses range values as observation inputs, exhibiting similar principles to those of the EKF [36]. As a result, position computations are no longer necessary, and the algorithm uses the range values directly for filtering. Since we can input the robot’s velocity both linearly and, in an angle, LPF + EKF can offer a more dynamic filtering method with greater accuracy than other approaches. Because LPF + EKF adds the robot’s velocity to predict the next trajectory, it is just a more promising process than LPF + KF. When used in noisy environments, it performs well, as shown in Algorithm 3.
**Algorithm 3** LPF + EKF1: 2: 3: 4:**Input: data** = ${[(x}_{raw}$,$\;y_{raw)},\;\left(x_{truth},\;y_{truth}\right),\;\left({\dot{x}}_{raw},\;{\dot{y}}_{raw}\right)]\;$ LPF–Extended Kalman filter (data) filtered_data = [] **For i in range (len(data));**5: Measurement (TOF ());6: LPF–Extended Kalman filter ()7: 
**{**8: 

**if** (Positions out of bound **then**9: 

**       return**$\left[(\hat{x},\;\hat{y}),\;(\dot{x},\;\dot{y})\;\right]$10: 

**else**11: 12: 13: 14: 15: 16: 17: 18: 19: 2 0: 21: 22: 

 LPF–Extended Kalman filter (); ∖∖calculate the expected robot’s position and velocity range State Estimate = $\left[\left(\hat{x},\;\hat{y}\right),\;(\dot{x},\;\dot{y})\right]$ Process Noise        ${\;z}_{t}=C_{t}x_{t}+W_{t},{\;W}_{t}\sim Ɲ\left(0,Q_{t}\right),$ Correction Step ${\;z}_{k}=H\;x_{k}+r_{k}$ Predict Step ${\;x}_{t}=g\left(x_{t-1},\;u_{t},\;v_{t-1}\right)$
${\;z}_{t}=h\left(x_{t},w_{t}\right)$ filtered_data return: filtered_data23: 

**end if**24: 
**}**25: LPF–Extended Kalman filter ();26: Print (filtered_data)27: **End for**

The suggested approach works well in particular indoor localization situations. As demonstrated by the suggested algorithm, LPF + KF performs well in high-noise environments and simple motions; LPF + MVG offers flexibility and resilience in a range of indoor environments; and LPF + EKF uses direct range values for precise position estimates.

## 6. Experimental Setup

### 6.1. Hardware Setup

The TurtleBot 3 robot, a Raspberry Pi onboard processor, a UWB tag module, and four POZYX anchors fastened to the experimental area’s sensor stand were the tools used in this experiment. TurtleBot 3, Raspberry Pi onboard processor, manufactured and sources by ROBOTIS Co., Ltd., headquartered in Seoul, South Korea and UWB sensor is manufactured by Pozyx Labs, located in Ghent, Belgium sourced from IRRI lab and KIMM, Korea. The anchors are connected to the computer via Ethernet cables and PoE switches. The computer has a UWB localization software Pozyx-creator-controller-2.1.0. package installed [37]. Table 1 contains a list of all the components used in the experiment.

### 6.2. Environment

**Figure 6:**
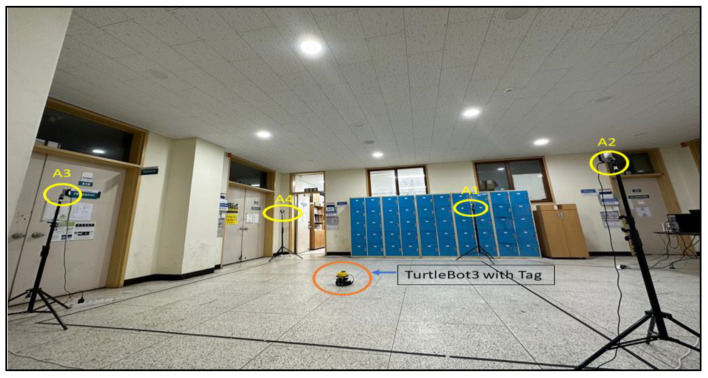
Indoor environment.

For operation, a sizable indoor space has been set up with a direct line of sight between the robot’s tag and the anchors. There are no big metal barriers or surfaces that reflect light nearby that could impede UWB signals. As seen in Figure 6, the area is well lit, enabling the robot to move around safely.

#### 6.2.1. Calibration

To obtain precise distance measurements between the anchors and the tag, the UWB localization was calibrated prior to the experiment’s commencement. The calibration is carried out using the ROS software package. Usually, taking measurements at different angles and distances between the tag and the anchors (A1–A4) is part of the calibration process [38].

#### 6.2.2. Localization

After UWB, the system was calibrated, and the localization algorithm was run on the computer to estimate the position of the robot in real time. The estimated position was then displayed on a 2D map of the environment and used for navigation tasks.

## 7. Experiment and Results

We created and carried out a thorough experimental scenario to evaluate the efficiency of the UWB indoor localization technology presented in this study. The studies were conducted with the tag and anchors in the best possible line of sight.

### 7.1. Experiment

We conducted three distinct trajectory path experiments (T1, T2, and T3), as illustrated in Figure 6. Trajectories T1, T2, and T3 represent square, circular, and free paths, respectively, with UWB tags positioned at fixed locations. Filtering techniques, including MVG, KF, and EKF, were employed to process the data. Subsequently, we applied our integrated techniques, combining MVG + LPF, KF + LPF, and EKF + LPF. The comparative analysis aimed to assess accuracy and precision by comparing the filtered data with the ground-truth positions. These results show valuable insights for optimizing UWB localization algorithms, emphasizing considerations such as accuracy and noise reduction.

### 7.2. Results

#### 7.2.1. Target 1—Square Path with (MVG, KF, EKF and MVG + LPF, KF + LPF, EKF + LPF) Filtering

**Figure 7:**
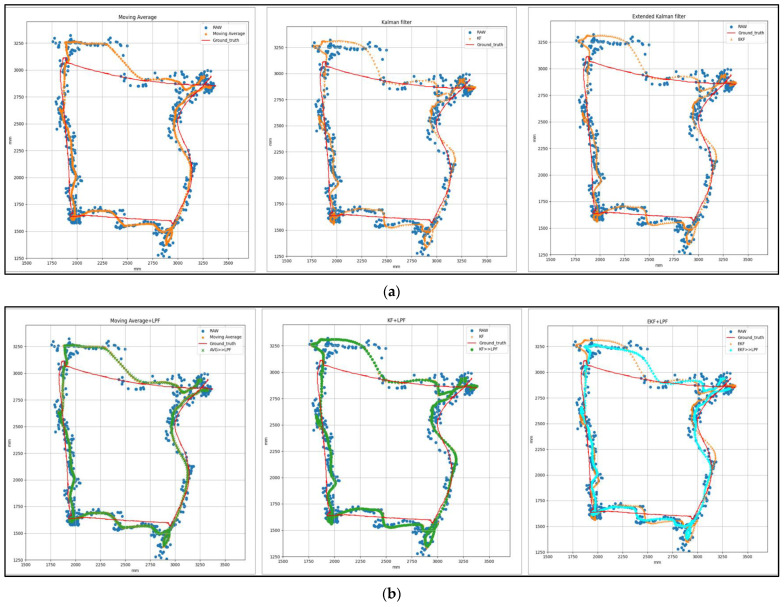
(**a**) Target 1—Square path with (MVG, KF, EKF) filtering. (**b**) Target 1—Square path with (MVG + LPF, KF + LPF, EKF + LPF) integrated filtering.

Figure 7 represents the T1 square trajectory path with (MVG, KF, EKF) and (MVG + LPF, KF + LPF, EKF + LPF) integrated filtering. The graph compares the raw and filtered data collected during the experiment to the ground truth. The trajectory moves at a speed of 0.5 m/s and covers a distance of 2 m. The positions along the X and Y axes, expressed in millimeters (mm), correspond to the collected data.

**Figure 8:**
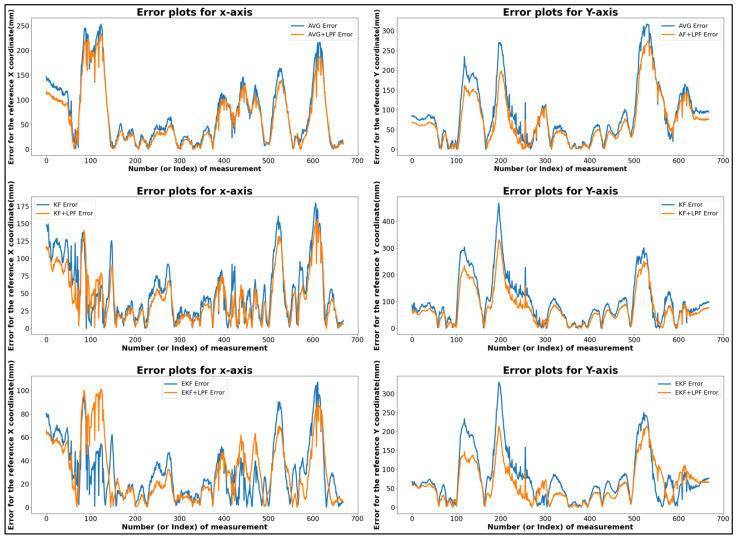
Error comparison graph of square trajectory path-T1 with (MVG, KF, EKF and MVG + LPF, KF + LPF, EKF + LPF) filtering.

Table 2 shows the data derived from the graphical representation in Figure 8. The table compares the square trajectory using (MVG, KF, EKF) filtering and the (MVG + LPF, KF + LPF, EKF + LPF) integrated filtering. Both trajectories move at a speed of 0.5 m/s and cover a distance of 2 m. Various measurements for these trajectories in terms of positions along the *X* and *Y* axes are outlined in the table.

The “trajectory with (MVG, KF, EKF)” had a maximum error of 180.82 mm along the *X* axis and 371.07 mm along the *Y* axis. The minimum error was 0.12 mm for both the *X* and *Y* axes. The absolute error difference between the maximum and minimum values, denoted as |Max.–Min.|, was 179.9 mm for the *X* axis and 371.85 mm for the *Y* axis. The mean error position was 52.19 mm for the *X* axis and 89.09 mm for the *Y* axis.

For the “Integrated filtering method with (MVG + LPF, KF + LPF, EKF + LPF)” of the square error trajectory, data were extracted from Figure 8. The maximum error along the X position was 163.81 mm, and along the Y position, it was 273.09 mm. The minimum error along the X position was 0.13 mm, and along the Y position, it was 0.09 mm. The absolute error difference between the maximum and minimum values was 163.68 mm for the *X* axis and 273 mm for the *Y* axis. The average error position was 46.6 mm for the *X* axis and 70.36 mm for the *Y* axis. These measurements provide valuable insights into the characteristics of the square trajectories, highlighting the range of positions, average positions, and the effect of the LPF on the data.

#### 7.2.2. Target 2—Circular Path with (MVG, KF, EKF and MVG + LPF, KF + LPF, EKF + LPF) Filtering

**Figure 9:**
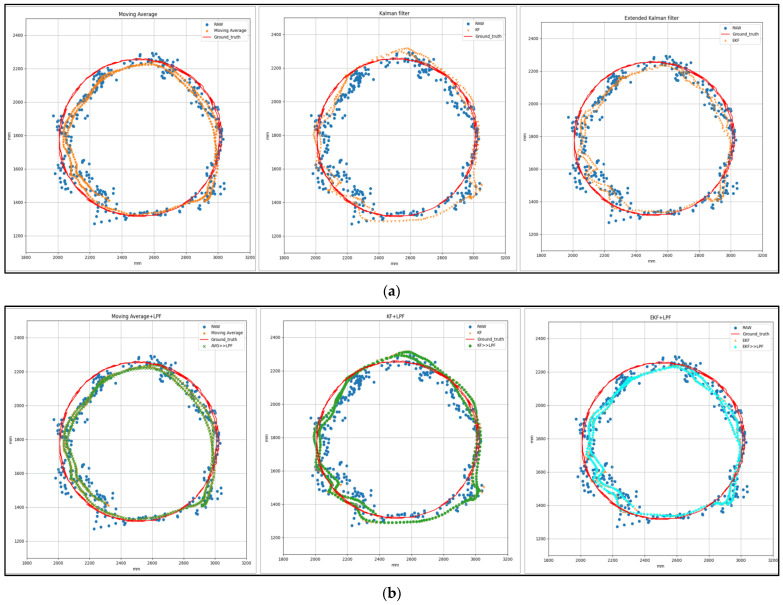
(**a**) Target 2—circular path with (MVG, KF, EKF) filtering. (**b**) Target 2—circular path with (MVG + LPF, KF + LPF, EKF + LPF) filtering.

Figure 9 shows the graphical measurement of the raw and filtered data obtained during the circular trajectory. The data were filtered using MVG, KF, and EKF filtering algorithms, as well as MVG + LPF, KF + LPF, and EKF + LPF integrated filtering algorithms. The trajectory spanned 2.2 m at a speed of 0.5 m per second. Table 3 presents the positions along the X and Y axes, expressed in millimeters (mm), that were matched to the data that were gathered.

Table 3 shows the “circular trajectory with (MVG, KF, EKF) filtering and (MVG + LPF, KF + LPF, EKF + LPF) with ground truth” data. Both trajectories covered 2.2 m at a speed of 0.5 m/s. When observing the “circular trajectory without integrated filter technique,” we found that the maximum error along the X position was 166.38 mm and the maximum error along the Y position was 341.05 mm. The minimum error along the X position was 0.46 mm, and along the Y position, it was 0.58 mm. The difference between the maximum and minimum values was 165.91 mm for the *X* axis and 340.47 mm for the *Y* axis. The mean error position was 56.34 mm for the *X* axis and 100.5 mm for the *Y* axis.

For the “circular trajectory with integrated filter technique” method, the maximum error along the X position was 158.51 mm, and the maximum error along the Y position was 286.22 mm. The minimum error along the X position was 0.52 mm, and the minimum error along the Y position was 0.81 mm. The difference between the maximum and minimum values was 157.99 mm along the *X* axis and 285.4 mm along the *Y* axis. The average error position along the *X* axis was 50.63 mm, and for the *Y* axis, it was 88.44 mm.

**Figure 10:**
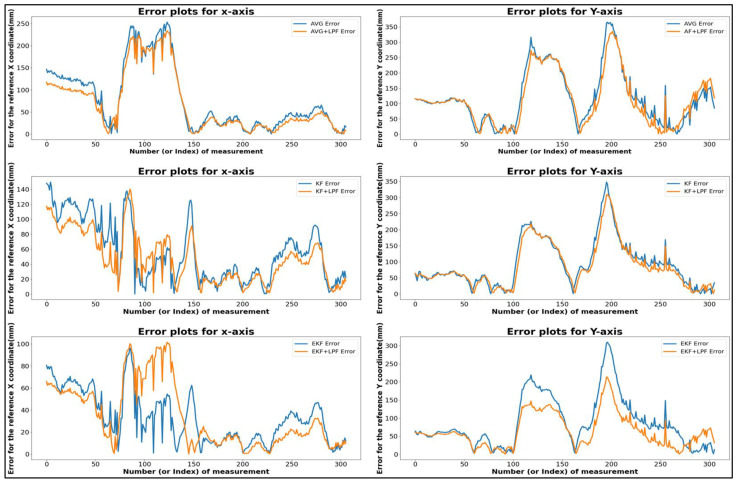
Error comparison graph of circular trajectory path-T2 with (MVG, KF, EKF and MVG + LPF, KF + LPF, EKF + LPF) filtering.

These calculated values provide valuable insights into the characteristics of the two filtering algorithms, revealing the range of positions, average positions, and the impact of the low-pass filter (LPF) on the data. The incorporation of the low-pass integrated filtering technique significantly enhanced the raw data, demonstrating improved accuracy in the trajectory data by effectively reducing errors and noise, as illustrated in Figure 10.

#### 7.2.3. Target 3—Free Path with (MVG, KF, EKF and MVG + LPF, KF + LPF, EKF + LPF) Filtering

**Figure 11:**
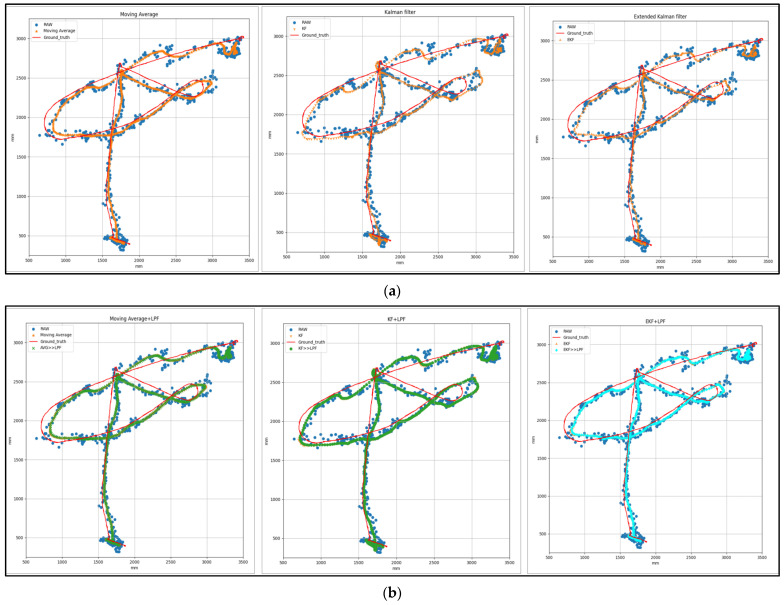
(**a**) Target 3—free path with (MVG, KF, and EKF) filtering. (**b**) Target 3—free path with (MVG + LPF, KF + LPF, EKF + LPF) filtering.

Figure 11 analyzed the performance of different filtering techniques for free trajectory data. Specifically, we compared the results of an integrated filtering technique and a non-integrated technique with a distance of 5 m and a constant velocity of 0.5 m/s.

We presented our findings in Table 4, which provides a detailed comparison of the error data for the free path trajectory with (MVG, KF, EKF) filtering and (MVG + LPF, KF + LPF, EKF + LPF) integrated filtering.

**Figure 12:**
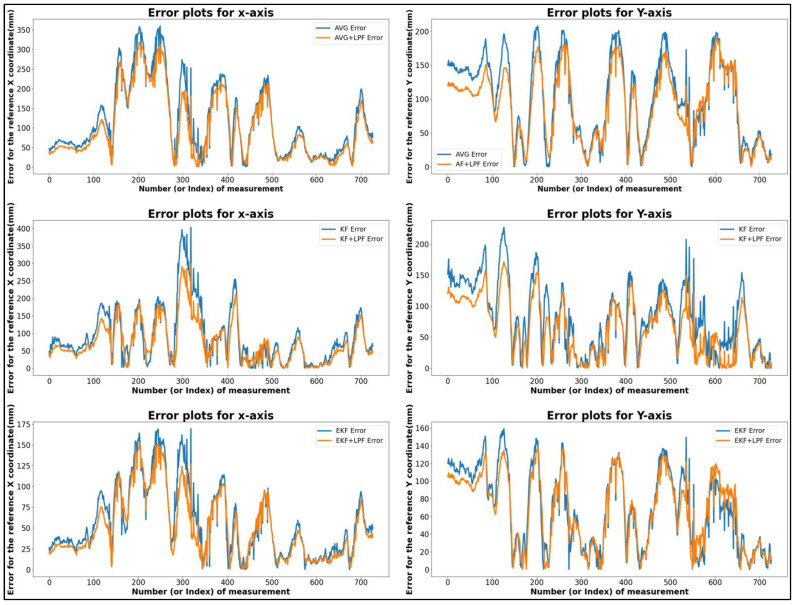
Error comparison graph of free trajectory path -T3 with (MVG, KF, EKF and MVG + LPF, KF + LPF, EKF + LPF) filtering.

This analysis showed that the maximum error along the X position was 310.84 mm, and the maximum error along the Y position was 197.99 mm, as shown by the graphical representation of (MVG, KF, and EKF) filtering in Figure 12. On the other hand, the minimum error was 0.20 mm along the Y position and 0.3 mm along the X position. Along the X and Y axes, the absolute difference error between the maximum and minimum values was 197.78 mm and 310.55 mm, respectively. According to the accompanying table, the average error position along the *X* axis was 88.84 mm, and along the *Y* axis, it was 84.36 mm.

Similarly, in the (MVG + LPF, KF + LPF, and EKF + LPF) integrated filtering graph, the maximum error along the X position was 256.74 mm, and the maximum error along the Y position was 166.50 mm. The minimum error along the X position was 0.74 mm, and along the Y position, it was 0.36 mm. The absolute error difference between the maximum and minimum values was 255.99 mm along the *X* axis and 166.13 mm along the *Y* axis. The mean position for the *X* axis was −41.72 mm, and for the *Y* axis, it was 22.81 mm. The average error position along the *X* axis was 76.25 mm, and along the *Y* axis, it was 74.07 mm.

It shows that adding a low-pass filter (LPF) to the raw filtered data can improve the accuracy of trajectory data, as seen in the results of the integrated filtering (MVG + LPF, KF + LPF, and EKF + LPF). Overall, our findings highlight the importance of using appropriate filtering techniques for trajectory data analysis, and the potential benefits of integrating LPF for improved accuracy.

#### 7.2.4. RMSE ABS Error and SD (Circular, Square, and Free Path)

**Figure 13:**
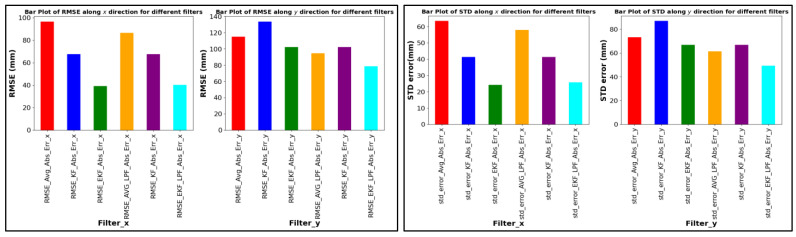
RMSE ABS error and SD (square path).

**Figure 14:**
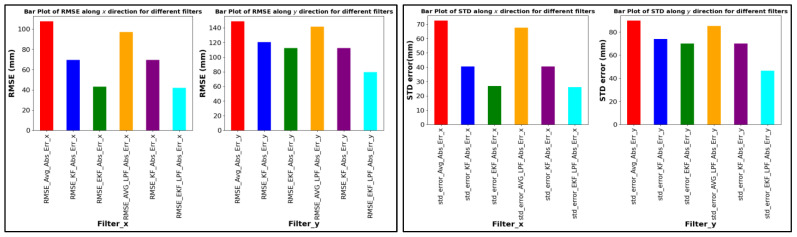
RMSE ABS error and SD (circular path).

**Figure 15:**
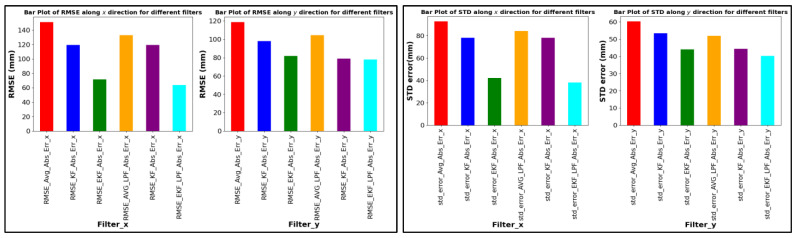
RMSE ABS Error and SD (free path).

Figure 13, Figure 14 and Figure 15 provide a comprehensive evaluation of different localization algorithms used in a UWB (ultra-wideband) localization system for three distinct trajectories: square path, circular path, and free path. The evaluation metrics include root mean square error (RMSE) and standard deviation (SD) for both X and Y coordinates.

Starting with the square path trajectory, the extended Kalman filter (EKF) algorithm demonstrates the lowest RMSE values for both X and Y dimensions, indicating superior accuracy in position estimation compared to other algorithms. The EKF also shows consistently low standard deviation values, which confirms the precision and reliability of the estimates, as displayed in Figure 13.

Moving on to the circular path, the EKF + LPF (extended Kalman filter with low-pass filter) combination emerges as the top performer with the lowest RMSE values in both X and Y directions. The combination’s effectiveness comes from integrating a low-pass filter, which improves the accuracy of the extended Kalman filter in tracking the circular trajectory and minimizing errors in position estimation. The competitive standard deviation values of EKF + LPF further emphasize stability in the estimated positions, as illustrated in Figure 14.

For the free path trajectory, the EKF + LPF configuration consistently outperforms other algorithms, displaying the lowest RMSE in both dimensions. The algorithm maintains its precision advantage, as indicated by lower standard deviation values, highlighting its efficacy in localizing UWB signals along a free and unrestricted path, as shown in Figure 15.

In summary, analyzing RMSE and SD values reveals that the EKF algorithm is excellent for the square path trajectory, while the EKF + LPF combination consistently outperforms other algorithms in the circular path and free path scenarios. These results provide useful insights when selecting the best algorithm based on trajectory characteristics and accuracy requirements in different applications.

#### 7.2.5. MAPE for Circular, Square, and Free Path

The mean absolute percentage error (MAPE) values represent the average percentage difference between the estimated and true positions in a UWB (ultra-wideband) localization system designed for an indoor environment.

According to Table 5, the extended Kalman filter with low-pass filter (EKF + LPF) has been shown to consistently outperform other algorithms in terms of position estimation accuracy, as evidenced by the MAPE analysis. Specifically, when following a square path trajectory, EKF + LPF records a minimal 1.18% MAPE for the *X* axis and an equally impressive 3.36% MAPE for the *Y* axis, indicating an average error of only 1.18% and 3.36%, respectively, in position estimation along the square trajectory. In contrast, alternative algorithms such as MVG, KF, and MVG + LPF exhibit higher MAPE percentages, indicating larger average percentage errors in position estimation.

Moving on to the circular path trajectory, EKF + LPF continues to perform exceptionally well, consistently reducing the percentage difference between estimated and true positions. It achieves a 1.76% MAPE for the *X* axis and a 3.30% MAPE for the *Y* axis, both of which are better than those of alternative algorithms.

Similarly, along the free path trajectory, EKF + LPF showcases the lowest MAPE percentages for both X and Y coordinates. Notably, it achieves a 3.59% MAPE for the *X* axis and a 3.47% MAPE for the *Y* axis, affirming accurate position estimation.

Overall, the MAPE data highlight that the extended Kalman filter with low-pass filter (EKF + LPF) consistently provides the most accurate position estimates across different trajectories in an indoor UWB localization system. The low MAPE percentages validate its effectiveness in minimizing errors and enhancing the precision of location estimation, making it a promising choice for applications requiring high accuracy in indoor positioning. The expression logic has been refined to better align with the core point of the paper, emphasizing the consistent superiority of EKF + LPF in position estimation across diverse trajectories.

#### 7.2.6. Overall Error % for Circular, Square, and Free Path

The “Overall Error %” in Table 6 presents a detailed analysis of the average error percentages associated with different localization algorithms used in an indoor UWB (ultra-wideband) localization system across three different trajectories—square path, circular path, and free path.

Among all the algorithms evaluated for the square path trajectory, the extended Kalman filter with low-pass filter (EKF + LPF) shows the most promising results, with an overall error percentage of 2.54%. This suggests that the EKF + LPF algorithm provides the most accurate position estimates on average when compared to other evaluated algorithms. The extended Kalman filter (EKF) also performs exceptionally well, even without the low-pass filter, with an overall error percentage of 2.86%. These results indicate that EKF-based algorithms, especially when combined with a low-pass filter, demonstrate superior accuracy in position estimation for square trajectory scenarios.

When it comes to the circular path trajectory, the EKF + LPF combination continues to lead, achieving an overall error percentage of 3.11%. The moving average filter (MVG) algorithm also shows competitive results, with overall error percentages of 6.70% and 6.08% for MVG with and without the low-pass filter, respectively.

For the free path trajectory, the EKF + LPF algorithm remains the most accurate, with the lowest overall error percentage of 4.88%. The EKF algorithm without the low-pass filter follows closely, with an overall error percentage of 5.32%. These results highlight the effectiveness of EKF-based algorithms, particularly when combined with a low-pass filter, in achieving accurate position estimates along free trajectories.

Table 6, which shows the overall error %, further strengthens the superiority of the extended Kalman filter with low-pass filter across all trajectories, offering the lowest overall error percentages. This information is useful for researchers and practitioners who are looking for the best algorithm to use in UWB localization systems in indoor environments, emphasizing the importance of considering the specific characteristics of different trajectories. The expression logic has been refined to emphasize the consistent superiority of EKF + LPF in position estimation across various trajectories.

#### 7.2.7. Comparison with Existing Methods

Table 7 shows a comparison of different methods for UWB indoor localization. Our proposed methods, the extended Kalman filter (EKF) and extended Kalman filter with low-pass filter (EKF + LPF), are particularly effective.

In comparison to research on “Reducing UWB Indoor Localization Error Using the Fusion of a Kalman Filter with a Moving Average Filter”, our strategy that uses a moving average filter (MVG), Kalman filter (KF), and KF with low-pass filter (KF + LPF) has competitive performance. Our Kalman ilter has an average error of 0.068 m, which is slightly higher than the 0.065 m reported in the cited study. However, when we incorporated a low-pass filter in our Kalman Filter + LPF configuration, the average error improved to 0.062 m. Similarly, our moving average algorithm produced a comparable average error of 0.064 m, and with the introduction of a low-pass filter, the error was further reduced to 0.054 m. Overall, our proposed approaches are more precise and exhibit smaller errors in UWB indoor localization when compared to existing methods. This marks a significant contribution to the field. We have revised the expression logic to clarify the comparison and highlight the improvements achieved by our proposed method.

## 8. Conclusions

In conclusion, our research has made significant progress in ultra-wideband (UWB) localization systems by introducing an integrated filtering approach that incorporates the low-pass filter (LPF) with the moving average (MVG), Kalman filter (KF), and extended Kalman filter (EKF). Through a comprehensive evaluation using a TurtleBot robotic platform and camera, we systematically investigated the performance of these filtering techniques in diverse trajectory scenarios, including square, circular, and free paths of varying distances. Our findings demonstrate the superiority of the extended Kalman filter with low-pass filter (EKF + LPF), particularly evident in the square path trajectory, where it exhibited the lowest root mean square error (RMSE) values of 40.22 mm (*X* axis) and 78.71 mm (*Y* axis). This integrated filtering method consistently outperformed MVG, KF, and EKF, resulting in reduced mean absolute error (MAE) percentages of 3.39% for square paths, 4.21% for circular paths, and 6.16% for free paths. The effectiveness of EKF + LPF stands out as a noteworthy contribution to accurate indoor localization for UWB systems.

Looking ahead, future research will focus on exploring additional filtering methods and integrating advanced technologies such as deep learning algorithms and sensor fusion to further enhance the precision and reliability of UWB localization systems in dynamic indoor environments. Additionally, the evaluation of probabilistic methods like particle filters, Bayesian filters, and support vector machines, considering their computational complexity, holds promise for addressing challenges in non-line-of-sight (NLOS) scenarios.

The presented integrated model can be practically deployed in various domains, including indoor navigation, asset tracking, and robotics. As we move forward, the continuous evolution of UWB positioning systems will benefit from ongoing efforts to refine location accuracy and ensure system reliability in diverse conditions. The demonstrated potential of EKF + LPF serves as a beacon for the continued progress and practical implementation of UWB localization systems across diverse applications.

## Figures and Tables

**Table 1:** Hardware specification.

Hardware Components	Description
UWB Localization System	POZYX UWB localization system Consists of UWB anchors and tags Operates in a frequency range of 3.5 GHz to 6.5 GHz Supports precise indoor positioning Range of up to 100 m
Camera	High-resolution RGB camera Minimum resolution of 1080 p Wide field of view (FOV) of at least 90° Interfaces with the computer/processing unit via USB 3.0
TurtleBot	TurtleBot 3 Burger model Equipped with a Raspberry Pi 4 single-board. Features a differential drive system with two DC motors Includes encoders for odometry calculations Equipped with a 360-degree LiDAR sensor ROBOTIS Co., Ltd., Seoul, South Korea.
Computer/Processing Unit	Intel Core i7 processor-based computer 8 GB RAM 256 GB SSD storage Runs on Ubuntu or ROS (Robot Operating System)
UWB Anchors and Tags	Four UWB anchor nodes One UWB tag
Power Supply	Input voltage: 100–240 V AC Output voltage: 12 V DC
Communication Interface	Gigabit Ethernet interface for high-speed data transfer Communication between the computer and processing unit, UWB system, and Turtle Bot
Mounting Hardware	In-house 3D printed mounting brackets and fixtures for the camera, UWB anchors, and tags

**Table 2:** Measurement error data for the square trajectory for both algorithms.

Trajectory	Distance	Speed	Max. (mm)		Min. (mm)		|Max.–Min.| (mm)		Mean (mm)	
			X	Y	X	Y	X	Y	X	Y
Square	2 m	0.5 m/s	180.82	371.07	0.12	0.12	179.9	371.85	52.19	89.09
Square (LPF)	2 m	0.5 m/s	163.81	273.09	0.13	0.09	163.68	273	46.4	70.36

**Table 3:** Measurement error data for the circular trajectory for both algorithms.

Trajectory	Distance	Speed	Max. (mm)		Min. (mm)		|Max.–Min.| (mm)		Mean (mm)	
			X	Y	X	Y	X	Y	X	Y
Circular	2.2 m	0.5 m/s	166.38	341.05	0.46	0.58	165.91	340.47	56.34	100.5
Circular (LPF)	2.2 m	0.5 m/s	158.51	286.22	0.52	0.81	157.99	285.4	157.99	285.4

**Table 4:** Measurement error data of free trajectory path for both algorithms.

Trajectory	Distance	Speed	Max. (mm)		Min. (mm)		|Max.–Min.| (mm)		Mean (mm)	
			X	Y	X	Y	X	Y	X	Y
Free	5 m	0.5 m/s	310.84	197.99	0.3	0.2	310.55	197.78	88.84	84.36
Free (LPF)	5 m	0.5 m/s	256.74	166.50	0.74	0.36	255.99	166.13	76.25	74.07

**Table 5:** MAPE % for circular, square, and free path.

Trajectory Square Path	MVG		KF		EKF		MVG + LFP		KF + LPF		EKF + LPF	
MAPE %	X	Y	X	Y	X	Y	X	Y	X	Y	X	Y
	2.82%	3.88%	2.04%	4.37%	1.18%	3.36%	2.50%	3.19%	1.71%	3.36%	1.20%	2.68%
Trajectory Circular Path	MVG		KF		EKF		MVG + LFP		KF + LPF		EKF + LPF	
MAPE %	X	Y	X	Y	X	Y	X	Y	X	Y	X	Y
	4.51%	4.39%	2.95%	3.56%	1.76%	3.30%	4.0%	4.16%	2.56%	3.30%	1.92%	2.38%
Trajectory Free Path	MVG		KF		EKF		MVG + LFP		KF + LPF		EKF + LPF	
MAPE %	X	Y	X	Y	X	Y	X	Y	X	Y	X	Y
	7.20%	5.54%	5.43%	4.36%	3.59%	3.47%	6.17%	5.05%	4.48%	3.37%	3.13%	3.51%

**Table 6:** Overall absolute percentage error, for circular, square, and free path.

Trajectory Square Path	MVG	KF	EKF	MVG + LFP	KF + LPF	EKF + LPF
Overall Error %	4.76%	4.22%	2.86%	4.09%	3.39%	2.54%
Trajectory Circular Path	MVG	KF	EKF	MVG+LFP	KF+LPF	EKF+LPF
Overall Error %	6.70%	4.73%	3.41%	6.08%	4.21%	3.11%
Trajectory Free Path	MVG	KF	EKF	MVG+LFP	KF+LPF	EKF+LPF
Overall Error %	9.97%	7.61%	5.32%	8.69%	6.16%	4.88%

**Table 7:** Table of comparison with existing methods.

Method	Comparison
Reducing UWB Indoor Localization Error Using the Fusion of a Kalman Filter with a Moving Average Filter [39]	Average Error (m)
	Kalman Filter = 0.065 m Moving Average = 0.057 m
Our Proposed Method (MVG, KF+LPF)	Kalman Filter = 0.068 m, Kalman Filter + LPF = 0.062 m Moving Average = 0.064 m, Moving Average + LPF = 0.054 m

## Data Availability

Data used in this paper can be made available by contacting the corresponding author, subject to availability.
